# Diffusion Weighted/Tensor Imaging, Functional MRI and Perfusion Weighted Imaging in Glioblastoma—Foundations and Future

**DOI:** 10.3389/fneur.2017.00660

**Published:** 2018-01-22

**Authors:** Gayle R. Salama, Linda A. Heier, Praneil Patel, Rohan Ramakrishna, Rajiv Magge, Apostolos John Tsiouris

**Affiliations:** ^1^Department of Neuroradiology, Weill Cornell Medical College, New York, NY, United States; ^2^Department of Neurological Surgery, Weill Cornell Medical College, New York, NY, United States; ^3^Department of Neurology, Weill Cornell Medical College, New York, NY, United States

**Keywords:** diffusion tensor imaging, glioblastoma, functional magnetic resonance imaging, diffusion-weighted imaging, perfusion-weighted imaging

## Abstract

In this article, we review the basics of diffusion tensor imaging and functional MRI, their current utility in preoperative neurosurgical mapping, and their limitations. We also discuss potential future applications, including implementation of resting state functional MRI. We then discuss perfusion and diffusion-weighted imaging and their application in advanced neuro-oncologic practice. We explain how these modalities can be helpful in guiding surgical biopsies and differentiating recurrent tumor from treatment related changes.

## Introduction

Advanced imaging is playing an increasingly more important role in the management of patients with neuro-oncologic disease. Its use can help with presurgical risk stratification and delineation of eloquent cortex. While the gold standard remains intraoperative mapping for identification of eloquent brain, preoperative imaging can be of immense value in understanding individual patient anatomy to help make surgery more efficient. In this way, advances in diffusion tensor imaging (DTI) and functional magnetic resonance imaging (fMRI) provide noninvasive means of brain mapping. Other modalities, like transmagnetic stimulation (TMS) are also useful adjuncts and can make intraoperative mapping more efficient. After surgery, advanced imaging can help distinguish between the historically vexing diagnoses of either tumor recurrence or treatment-related change. In this review, we focus on the presurgical utility of DTI and fMRI and then move toward discussing how perfusion and diffusion imaging can more effectively guide patient management in diagnostically challenging situations.

## Diffusion Tensor Imaging

Diffusion tensor imaging (DTI) has provided the first *in vivo* visualization of white matter tracts in the brain (1). This fiber tracking technique has become an essential component of a multimodality approach to presurgical intraoperative planning and decision making. It demonstrates morphologic and anatomic information, previously inaccessible to the neurosurgeon without direct electrical stimulation, including demonstration of the corticospinal tract (CST) and arcuate fasciculus, which can influence the extent of surgical resection. As asserted by Potgieser et al., “the ultimate goal and major challenge in glioma surgery is to obtain maximal resection while minimizing loss of neurological function,” and DTI provides a means to protect eloquent white matter tracts (1).

**Figure 1:**
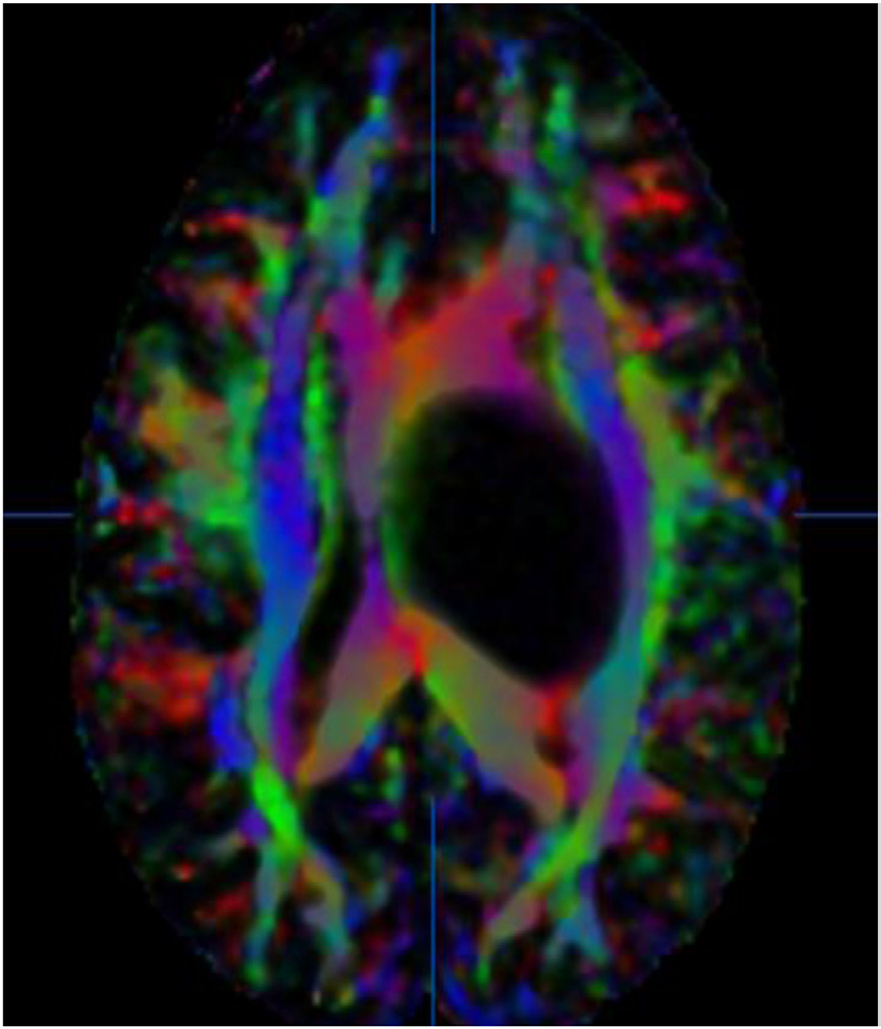
36-year-old woman with a left thalamic glioblastoma. Fractional anisotropy (FA) color-coded map with tracts coded in blue for the superior–inferior plane, red for the horizontal plane, and green for the anterior–posterior plane. No copyright permissions were required to use these images.

**Figure 2:**
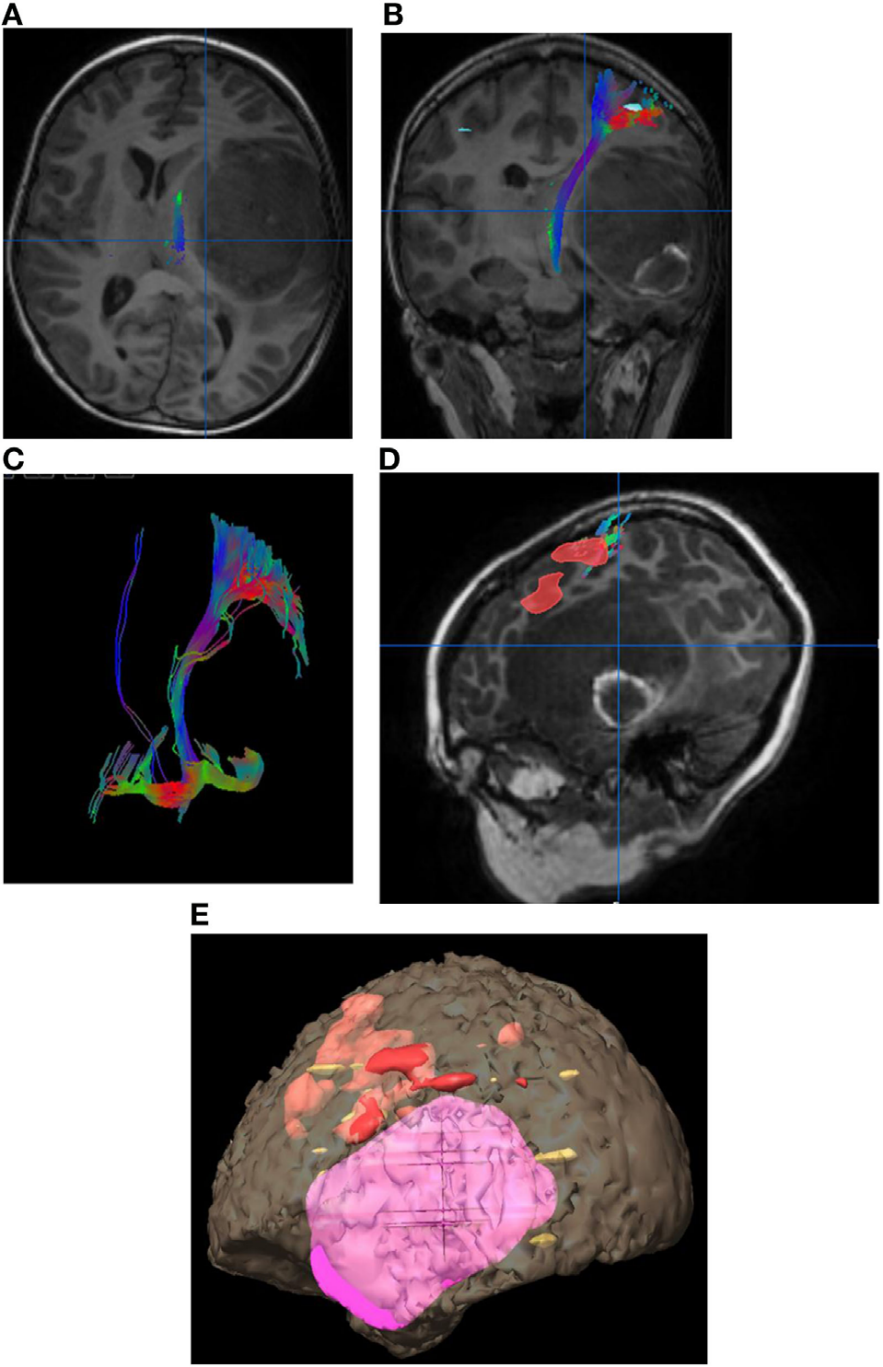
Eight-year-old right-handed boy with left temporal lobe glioblastoma. **(A)** Tractography in the axial plane demonstrating the left corticospinal tract (CST). **(B)** Tractography of the left CST in the coronal plane. **(C)** Three-dimensional fiber tracking of the left CST. **(D)** Sagittal T1-weighted image with fused language activation colored red demarcating Broca’s area, which is elevated by the left temporal lobe tumor. **(E)** Three-dimensional reconstruction with the tumor in pink and task-based functional areas of eloquent language in red, yellow, and orange. No copyright permissions were required to use these images.

**Figure 3:**
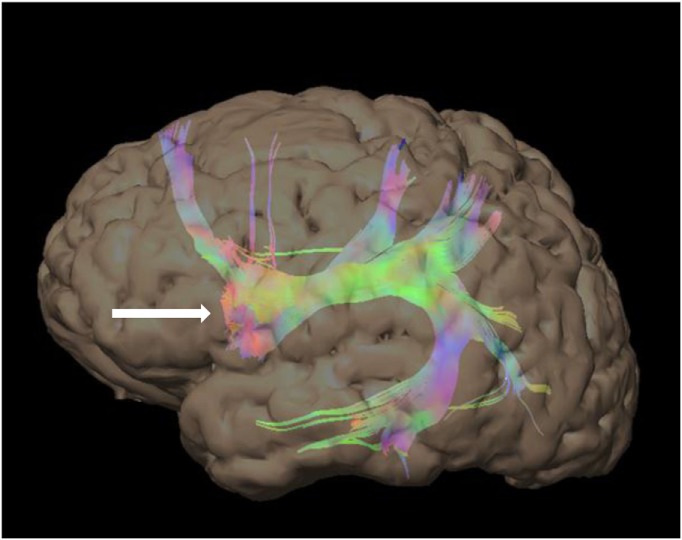
48-year-old right-handed patient with a right-sided temporal lobe glioma with left hemisphere dominance. Three-dimensional color tractography of the arcuate fasciculus from a task-based functional area consistent with Broca’s area (red blob marked with white arrow) viewed from the left. No copyright permissions were required to use these images.

**Figure 4:**
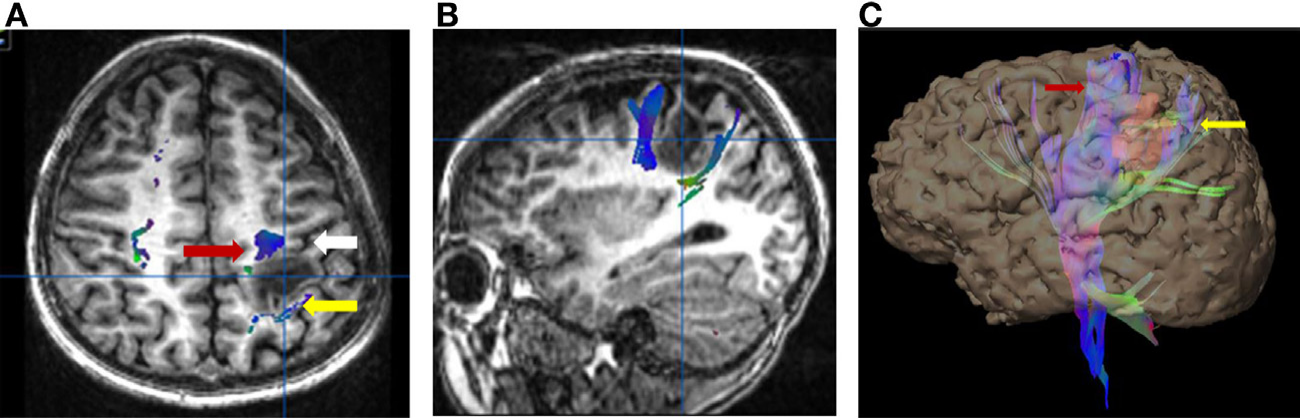
Six-year-old right-handed boy with WHO grade III anaplastic astrocytoma in the subcortical white matter of the left postcentral gyrus. Central sulcus is marked with a white arrow. **(A)** Axial T1-weighted anatomic image overlaid with directionally color-coded fiber tracking demonstrating the motor (red arrow) and sensory (yellow arrow) corticospinal tracts spread apart by the lesion. **(B)** Sagittal T1-weighted anatomic image overlaid with directionally color-coded fiber tracking demonstrating the motor (red arrow) and sensory (yellow arrow) corticospinal tracts spread apart by the lesion. **(C)** Three-dimensional reconstruction with the tumor, colored red, separating the motor (red arrow) and sensory fiber tracts (white arrow). No copyright permissions were required to use these images.

By measuring the diffusivity of water molecules, a map of the axonal network in the brain is created. Specifically, DTI uses anisotropy, the restriction of random three-dimensional (3D) Brownian motion of water molecules in white matter, to estimate the *in vivo* axonal direction within a particular voxel (2, 3). Tractography pieces together this information from voxel to voxel to model long-range pathways of white matter tracts (4). DTI, developed from diffusion-weighted imaging (DWI), uses magnetic gradients in at least six directions to create a model of diffusion in three dimensions (5). The direction of maximum diffusivity of water molecules coincides with the main white matter fiber orientation. Fractional anisotropy (FA), a unit-less numerical value between 0 and 1, correlates with the degree of directionality of diffusion, with higher FA values corresponding to greater directionality. These FA values can be used to create color-coded tractography maps, with blue corresponding to tracts traveling in the superior-inferior plane, red for tracts in the horizontal plane, and green for tracts in the anterior–posterior plane (Figure 1) (6, 7). Another way of visualizing the data is through fiber tracking, which demonstrates neural tracts in 3D. For example, DTI has been used to interrogate pathways, such as the CST (Figures 2 and 4), optic tract, superior longitudinal fasciculus, and arcuate fasciculus (Figure 3) (1, 5, 8–11).

In the presence of tumor, white matter can be displaced, disrupted, edematous, or infiltrated by tumor. DTI can demonstrate the local effects of tumor on white matter integrity. Four patterns have been typically described: (1) normal signal with altered position/direction suggesting tract displacement; (2) decreased but present signal with normal direction and location suggesting vasogenic edema; (3) decreased signal with disruption of fiber tracts suggesting tumor infiltration; and (4) loss of anisotropic signal suggesting tract destruction (5).

**Figure 5:**
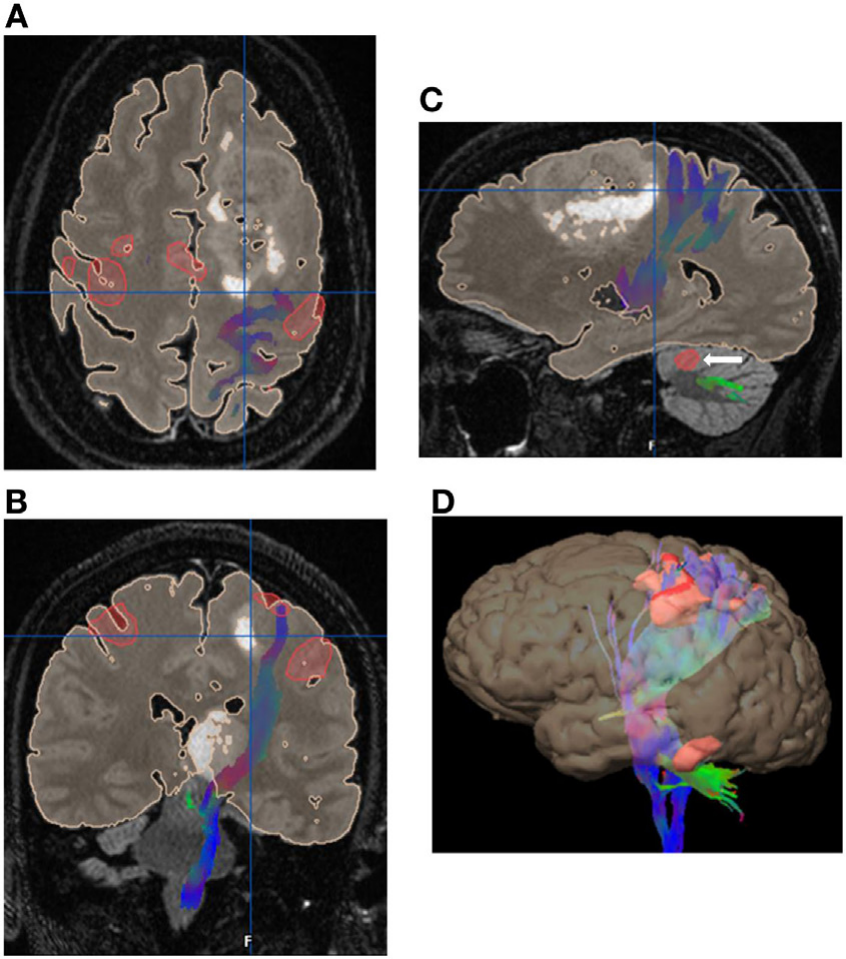
52 years old with gliomatosis involving the left posterior frontal lobe and left thalamus. Integrated surgical plan with task-based functional motor activation with the associated corticospinal tract fiber tracking. **(A)** Axial image with the eloquent motor cortex in red with the associated left corticospinal tract (CST) fiber tracking (blue) displaced posteriorly by the left frontal lobe mass. **(B)** Coronal image with the eloquent motor cortex in red with the associated left CST fiber tracking (blue) displaced laterally by the left thalamic mass. **(C)** Sagittal image with the left CST fiber tracking (blue) displaced posteriorly by the left frontal lobe mass. Note the associated cerebellar activation (white arrow). **(D)** Three-dimensional reconstruction with eloquent motor cortex in red and directionally color-coded CST fiber tracking. No copyright permissions were required to use these images.

Diffusion tensor imaging provides a presurgical and intraoperative tool, that in conjunction with other modalities, enables a safer, more patient-specific procedure (12). Integration of DTI/fMRI into neurosurgical navigation systems to provide patient-specific guidance will contribute to the efficacy and safety of neurosurgical resections, especially in the presence of anatomic distortion (Figure 5) (13, 14).

Tractography requires a diffusion model, a fiber-tracking algorithm, and a set of anatomical regions of interest (ROIs). The fiber-tracking algorithm reconstructs the trajectory of the white matter based on the directional information given by the diffusion model and the anatomical ROIs. The fiber-tracking algorithm may use deterministic, probabilistic, filtered, or global approaches which are all different mathematical analyses of the same DTI pulse sequence. An exact knowledge of white matter tracts is required to identify errors generated by these algorithms (15).

Concordance between DTI tractography and direct electrical stimulation is reportedly high with a sensitivity of 92.6% and specificity of 93.2% (1, 16). Furthermore, improved outcomes have resulted when preoperative DTI fiber tracking has been performed with a decrease in postoperative deficits from 32.8 to 15.3% and a longer median survival in high-grade glioma patients of 21.2 months compared to 14.0 months in the control group (17).

Although a promising modality for improved surgical planning and patient safety, DTI is subject to several pitfalls and limitations. First, the lack of a standard analysis protocol limits reproducibility and accuracy (15). Second, DTI has limited accuracy in the presence of crossing fibers and false tracts may be created. Third, the DTI sequence is very susceptible to inhomogeneity within the magnetic field. This limiting factor is magnified in the intraoperative setting because of increased susceptibility from air-tissue boundaries (1). Fourth, tumor involvement can change the architecture of white matter, so DTI may underestimate the presence of functional white matter tracts in the presence of tumor (18). Fifth, there is a high degree of user discretion required in determining appropriate FA thresholds made more complicated by tumor-related distortion and edema; however, ongoing improvements in software allowing for automatic segmentation can help practitioners separate noise and artifact from real white matter tracts. Finally, brain shift during the course of surgery from positioning, anesthesia, retraction, edema, and CSF leakage can result in loss of spatial congruency between cerebral structures and images, limiting tractography accuracy (19, 20). Since intraoperative parenchymal shift may be up to 15 mm, a recommended safety margin of at least 5–10 mm should be taken into account when approaching eloquent tracts (19, 21–23). However, such suggestions of “margin” are at best guides; the safest approach is to integrate intraoperative subcortical mapping with navigational data including DTI. Advents in technology including ultrasound guided reorientation of navigational data have emerged, but none are exacting enough to provide the spatial resolution necessary to operate near eloquent white matter tracts.

Many authors feel that DTI is no longer the gold standard for 3D fiber tracking produced by DWI (24–26). Currently, most DTI sequences utilized for intraoperative mapping use 64 or fewer diffusion directions with only a single shell of diffusion values (*b* = 1,000 s/mm^2^) and an approximate 5–6-min acquisition. These technical factors cannot overcome the serious crossing fiber problem. DTI fails to resolve the multiple fiber directionalities per voxel limitation required to visualize smaller white matter tracts or the corticospinal tract in its true entirety (24). Despite the advent of higher order models such as high angular resolution diffusion imaging (HARDI) and diffusion spectrum imaging (DSI) which produce more robust and accurate fiber orientation, newer methods have not yet translated well into the clinical arena due to complex post-processing algorithms and long acquisition times (27, 28). The first clinical use of advanced fiber tracking methods was a prospective study, which successfully identified language tracts of glioma patients preoperatively and predicted postoperative functional recovery with HARDI q-ball fiber tractography (27).

Recent advances in MR technology including simultaneous multislice echoplanar imaging, multiband excitation, and the use of multiple receivers has accelerated acquisition times permitting DSI to be used clinically. Many of these developments came about due to the work being done on the human connectome project (28). The latest entry to map white matter fibers is diffusional kurtosis imaging (DKI), which is qualitatively comparable to DSI. DKI has a shorter scan time and is therefore a potential clinical favorite (29).

Diffusion tensor imaging is not sufficiently accurate for ideal surgical planning, but it remains to be seen which diffusion-weighted method, HARDI, DSI, or DKI, will achieve clinical predominance in the future. All of these methods may overcome current DTI limitations and provide more reproducible and accurate fiber tracking.

## Functional MRI

Functional MRI is a technique to detect eloquent cortex by identifying increased blood oxygen levels in areas of the brain that are activated by task-based paradigms. Optimal presurgical planning requires maximal resection and minimal deficits in areas of eloquent cortex, particularly those contributing to motor and language function. Task-based fMRI correlates with electrophysiology, Wada testing and prediction of functional loss postoperatively (30–33). fMRI indirectly measures neuronal activity by looking at areas of increased blood flow with a specific pulse sequence that uses the ratio of oxyhemoglobin to deoxyhemoglobin as a contrast agent, also known as blood oxygen level-dependent (BOLD) imaging (34).

**Figure 6:**
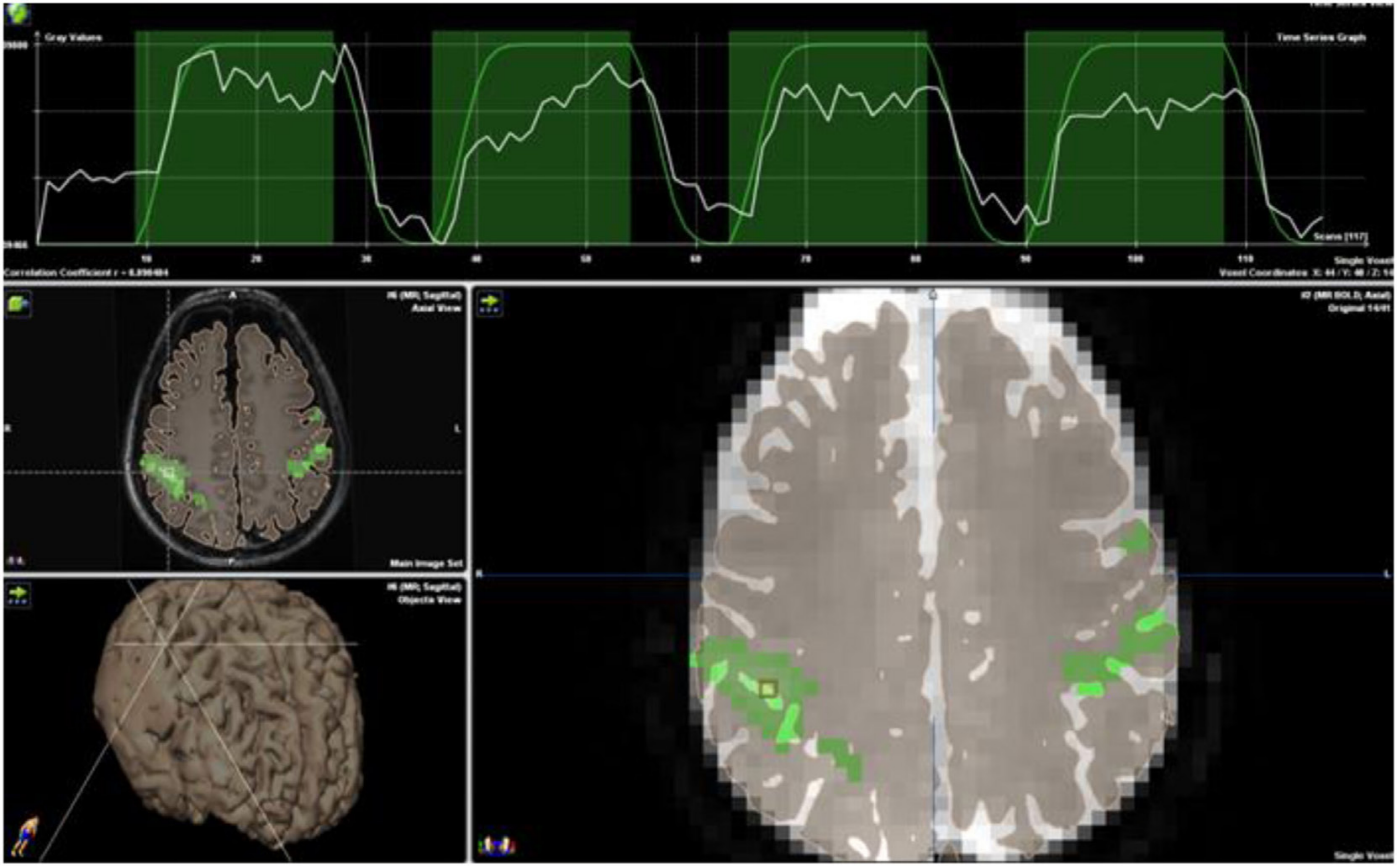
Block paradigm for bilateral finger tapping demonstrating eloquent cortex marked in green. Note the time course strip at the top of the image. Blood oxygen level-dependent activation (white line) is overlaid onto the block paradigms’ design (green line) of 18 s of rest followed by 36 s of activation, repeated three times for a total paradigm length of 4 min. No copyright permissions were required to use these images.

For surgical mapping, subjects alternate between a passive resting state and an active task-performing state, using the so called “block” paradigm design (Figure 6) (35). An arbitrary statistical threshold determines which voxel is considered “active” and setting the correct threshold is key to limiting noise and optimizing sensitivity (35). Sometimes a scoring system, the Laterality Index is used to choose the dominant hemisphere comparing the total number of active voxels on each side (36). Based on tumor proximity to an eloquent area, approximately 20% of total neurosurgical cases require fMRI mapping.

Functional MRI motor mapping correlates extremely well with functional areas identified by direct cortical stimulation (DCS) with fMRI having a sensitivity and specificity of 95–100% (37–39). In contrast, fMRI language mapping is less robust with sensitivity and specificity ranging between 37–91 and 64–83%, respectively (38). Nevertheless, fMRI is rapidly becoming the study of first resort for determining language dominance in the preoperative setting, as a non-invasive alternative to the Wada test. The Wada test, an intracarotid amobarbital procedure, is currently considered the gold standard. However, it is invasive, may lead to more permanent deficits in vascular compromised patients, has reversible side effects that can temporarily distress patients, must be performed rapidly (3–5 min), and can give unreliable results with variations in vascular anatomy. fMRI is cheaper, repeatable, and often less distressing to the patient than Wada testing (35).

Studies comparing the extent of tumor resection with fMRI verses without are limited as most are retrospective. In the few prospective studies that exist the extent of resection in high-grade gliomas, the functional status at 6 months, and progression-free survival (PFS) were improved with DTI functional neuronavigation (17, 39–41).

Limitations to fMRI include the presence of MRI contraindications, such as pacemakers, severe obesity, and claustrophobia, as well as lack of attention and inability to follow task-related commands. The output of task-based fMRI is highly dependent on adequate task performance (42). Additionally, task-based fMRI requires trained personnel and postprocessing, which are difficult to standardize (42, 43). Susceptibility artifact from blood products or metallic artifact from surgical plates and dental work may also affect the BOLD sequence used in fMRI. The abnormal vascularity found in and around high-grade gliomas may interfere with the BOLD signal resulting in false-negative results. Likewise, perilesional edema and brain plasticity may contribute to false-positive activations (44).

**Figure 7:**
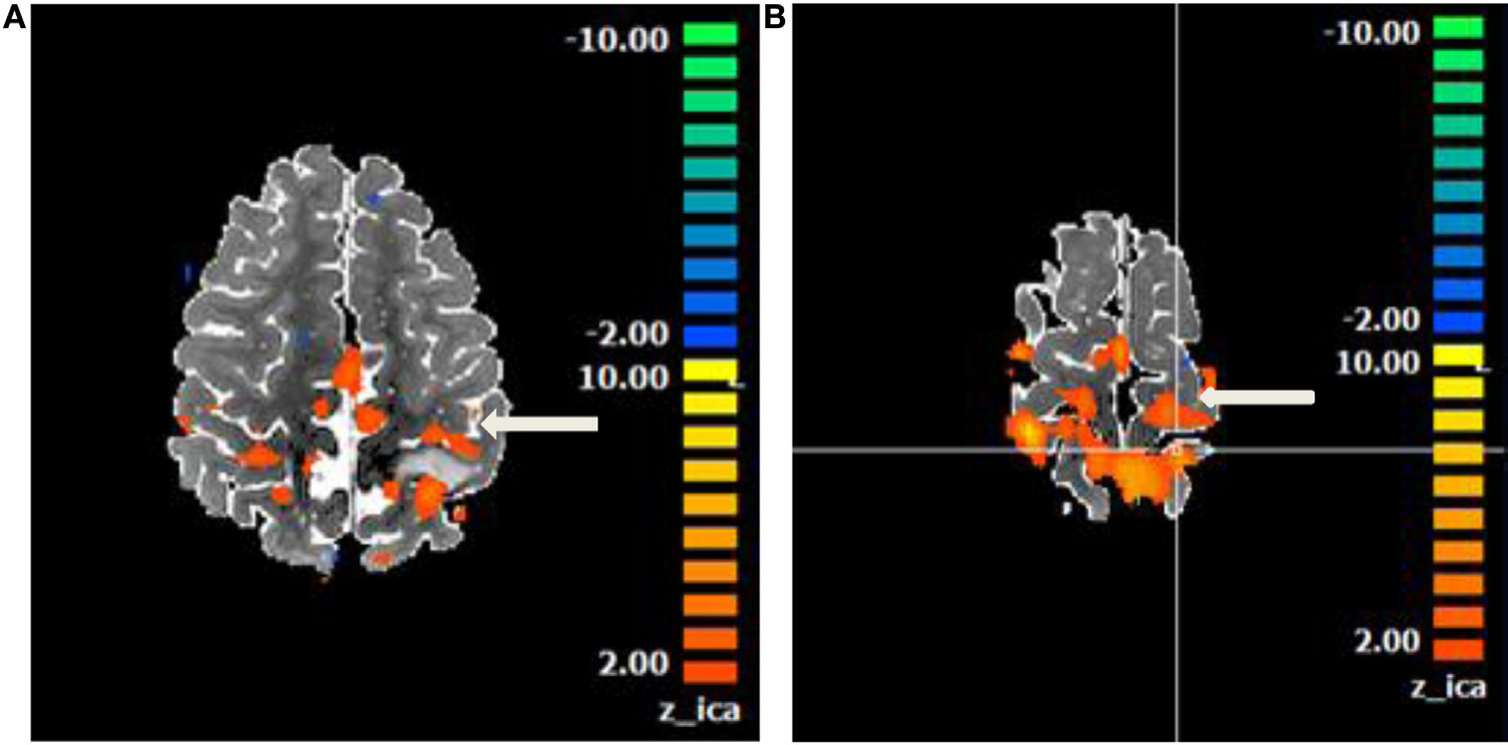
Motor network identified on a resting state functional magnetic resonance imaging in an anesthetized 6-year-old demonstrating eloquent cortex on both sides of a left postcentral gyrus glioma. Central sulcus is marked with white arrow. **(A)** Axial T2 image with overlaid motor network, in red, separated by T2 hyperintense glioma. **(B)** Axial T2 image with overlaid motor network on an image slice immediately superior to image **(A)**. No copyright permissions were required to use these images.

In patients who are unable to follow commands, an alternative to task-based fMRI is resting-state fMRI (rs-fMRI) (45). Rs-fMRI uses endogenous brain activity, detectable with the BOLD sequence, to identify areas that are interacting at rest to delineate distinct functional networks (46). Rs-fMRI generates correlation maps that are similar to functional maps obtained from task activation (47). Patient participation is not required and fluctuations in BOLD persist under conditions of sleep and anesthesia as well as in the presence of tumors (Figure 7) (48–50). In theory, rs-fMRI can be used in patients of any age and of any cognitive ability. Rs-fMRI is dependent on the selection of a “seed” region in a characteristic location (i.e., the hand-motor area to identify the sensorimotor network) and is thus biased by the technical limitations of the operator (51). Nevertheless, resting-state studies can salvage exams in uncooperative (pediatric or adult) and obtunded patients. While still experimental, increasing numbers of clinical studies proving concordance with task-based fMRI or intraoperative mapping have been published validating the clinical use of rs-fMRI (52–55).

Ultimately, fMRI cannot delineate what exact cortical area is required for a specific function. That said, it can help focus the mapping procedure intraoperatively to improve surgical efficiency. Moreover, it can be helpful in lateralizing language in right-dominant patients. As with DTI though, fMRI requires extensive postprocessing and institutional resources to operationalize on a regular basis. Hospitals without these resources can still perform excellent, safe surgery with DCS. Future directions for fMRI may include its use as an adjunct in planning radiation doses to minimize the effects of radiation toxicity or its use in planning safe trajectories and heat maps for patients who undergo laser interstitial thermal therapy. Finally, more widespread incorporation of ultra-high field magnets (7 T) will help improve the mesoscopic resolution of fMRI, particularly as it relates to BOLD imaging (56).

## MR-Perfusion and DWI

Magnetic resonance perfusion-weighted imaging (PWI) and DWI provide information on tumor physiology that is not accessible with conventional sequences. PWI methods include dynamic susceptibility contrast (DSC) and dynamic contrast-enhanced (DCE) perfusion. DSC generates hemodynamic parameters such as relative cerebral blood volume (rCBV) that theoretically reflects microvascular density or area (57). DCE generates a similar parameter, plasma volume (Vp), and K-trans, which is a marker of microvascular permeability or capillary “leakiness.” DCE has gained more interest recently due to better spatial resolution and less susceptibility artifacts than DSC, an important attribute in the postoperative setting or in hemorrhagic masses. An alternate sequence first used in the study of ischemia, DWI provides a visual representation of molecular water motion with areas of reduced diffusion [low apparent diffusion coefficient (ADC)] correlating with increased tumor cellularity and other factors (57). In the pretreatment setting, PWI and DWI have been studied as tools to help narrow the differential diagnosis, grade gliomas, guide biopsies, and assess prognosis and potential treatment success.

As neoplastic and inflammatory conditions with significant differences in management can share conventional imaging findings, PWI and DWI may be of use in narrowing the differential diagnosis. For example, high-grade gliomas demonstrate relatively increased rCBV in the peritumoral T2 hyperintense region compared to metastases, which may be related to surrounding infiltrative non-enhancing tumor. In addition, assessment of the DSC signal intensity curve has shown significant differences between high-grade glioma and metastasis in the contrast-enhancing region as well as the peritumoral region (58). Primary CNS lymphoma can also mimic a high-grade glioma but demonstrates lower rCBV and ADC values (59–61). Analysis of the DSC signal intensity curve has demonstrated significant differences between lymphoma, high-grade glioma, and metastasis (62). Certain non-neoplastic pathologies such as tumefactive demyelination similarly demonstrate lower rCBV values (63).

**Figure 8:**
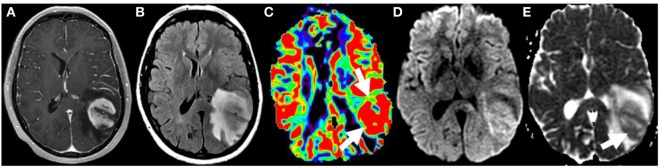
Glioblastoma on initial presentation. T1W postgadolinium **(A)** and T2W FLAIR **(B)** magnetic resonance sequences demonstrate a large intra-axial heterogeneously enhancing left parietal mass with extensive surrounding edema and/or infiltrative neoplasm. Elevated relative cerebral blood volume (arrows) is noted on the dynamic susceptibility contrast perfusion map **(C)** compatible with increased vascular density. The diffusion-weighted imaging **(D)** and the corresponding apparent diffusion coefficient map **(E)** demonstrate heterogeneous restricted water motion (arrow) within this mass most compatible with hypercellularity. No copyright permissions were required to use these images.

There is a strong correlation between elevated rCBV and high-grade gliomas (Figure 8), with an important exception being low-grade oligodendrogliomas, which can demonstrate relatively elevated values (64–66). Similar correlation has been found with higher K-trans (67, 68) and Vp (69). Lower ADC values have also been reported in higher grade gliomas, related to their increased cellularity (64, 70). Low-grade gliomas undergoing transformation can demonstrate increased rCBV up to 12 months before contrast enhancement is apparent on conventional imaging (71). If this finding were confirmed, PWI would be important to include in surveillance imaging of low-grade gliomas. Starting or changing treatment based on this information and whether early treatment leads to better oncologic outcomes would also need to be debated. However, as PWI and DWI measurements cannot completely obviate the need for tissue sampling at this time, their utility may be greater in the selection of biopsy targets to reduce undersampling (72–74).

The prognostic and predictive capabilities of PWI and DWI in the pretreatment setting have been assessed in several studies related to glioblastoma. Elevated baseline rCBV and K-trans values correlated with worse overall survival (OS) after cytotoxic therapy (75–77). Reduced ADC values prior to cytotoxic therapy were associated with reduced OS (78, 79). Reduced ADC values were also associated with worse outcomes in a multicenter study of recurrent glioblastoma treated with bevacizumab (80). On the contrary, in treatment-naive glioblastoma, reduced ADC values before antiangiogenic therapy correlated with better outcomes, possibly due to an association with MGMT methylation, highlighting the genetic differences of untreated and recurrent glioblastomas (81). However, the relevance of these findings remains unclear with a recent study suggesting that clinical parameters outperformed advanced imaging metrics in predicting survival at time of diagnosis (82).

In the posttreatment setting, MRI has an essential role with PWI and DWI providing potentially additive information about early therapeutic response with both standard and antiangiogenic therapies and differentiating response from pseudoresponse and true progression from treatment changes. For example, Mangla et al. showed that an increase in rCBV at 1 month after therapy was predictive of poor 1-year survival (median survival 238 vs. 529 days), whereas change in tumor size was not predictive (83). In a recent meta-analysis, Choi et al. qualitatively reviewed 13 studies and pooled-hazard ratios with rCBV as the marker for responders and non-responders, concluding that PWI could be considered as a predictive or prognostic biomarker in patients treated with a bevacizumab-based regimen (84). PWI may therefore potentially overcome the limitation of pseudoresponse on conventional postcontrast imaging after antiangiogenic therapy. PWI (and DWI) may additionally help to increase sensitivity and specificity for non-enhancing tumor, given the non-specificity of T2 changes after bevacizumab treatment (85, 86).

Diffusion-weighted imaging-derived metrics have similarly shown prognostic potential in the posttreatment setting. For example, Rahman et al. evaluated changes in ADC between baseline and posttreatment exams in recurrent glioblastoma patients on bevacizumab alone or in combination with other chemotherapies. Using histogram analysis, which better characterizes the distribution of values within the area of concern, ADC parameters from baseline and 3- to 6-week posttreatment exams stratified overall and PFS (87). The timing of evaluation appears to have an impact as noted in a study of 37 glioblastoma patients treated with standard therapy followed by adjuvant temozolomide and an antiangiogenic drug. Changes in diffusion parameters were assessed pre-, mid-, and postradiation therapy and correlated with 6-month PFS. Changes in ADC from mid- to postradiation were more significant than other time points, a notable finding as a mid-therapy exam is not typically performed (88).

**Figure 9:**
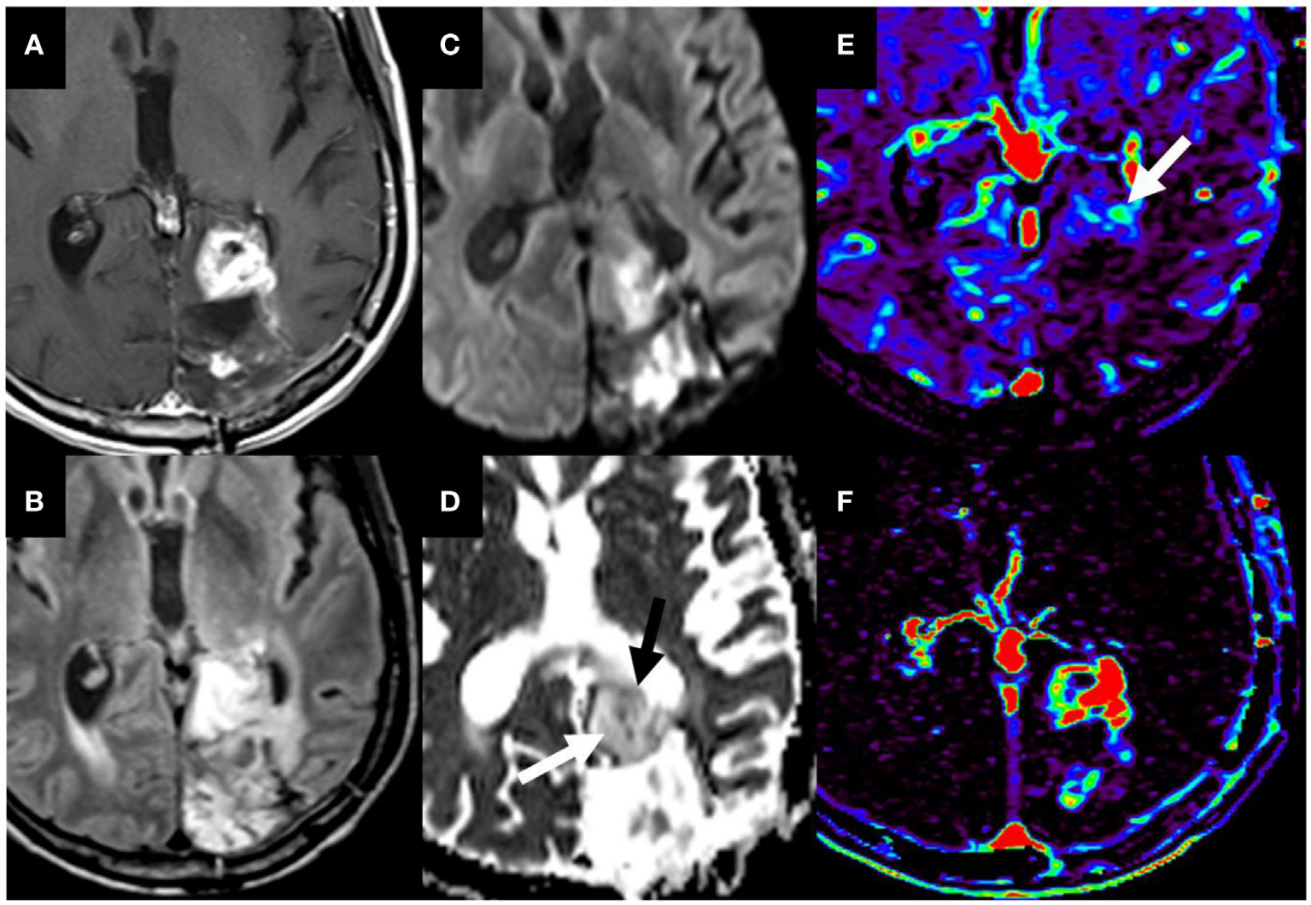
T1W postgadolinium **(A)** and T2W FLAIR **(B)** magnetic resonance (MR) sequences demonstrate a region of heterogeneous mass-like enhancement at the anterior border a glioblastoma resection cavity in the left occipital lobe that had progressed since the prior study. Diffusion-weighted imaging **(C)** and the corresponding ADC map **(D)** demonstrate predominately increased water motion suggesting necrosis [**(D)**, white arrow]. The dynamic susceptibility contrast maps demonstrate predominately decreased microvascularity on the Vp map **(E)** with markedly increased permeability on the K-trans map **(F)**. However, there is a smaller internal region of restricted diffusion [**(D)**, black arrow] and increased Vp [**(E)**, white arrow]. The combination of these imaging findings, diffusion and perfusion metrics are most compatible with pseudoprogression/treatment changes with a smaller internal region of recurrent tumor. These findings were confirmed on pathology. No copyright permissions were required to use these images.

Perfusion-weighted imaging has also shown promise in helping to differentiate tumor from radiation necrosis or pseudoprogression (Figure 9). A recent meta-analysis calculated pooled sensitivities and specificities of 90% and 88% and 89% and 85% for identifying recurrent tumor with DSC and DCE, respectively (89). The most commonly evaluated DSC perfusion parameter has been rCBV with consistent differences shown between tumor and treatment change (90–93). Exceptions include Song et al. who found a difference using ADC but not rCBV (94) and Kong et al. who found a difference only in the MGMT methylated group (95). Both quantitative and semiquantitative DCE approaches have been increasingly evaluated in the literature and have demonstrated success in separating tumor and treatment change. For example, Thomas et al. evaluated the 90th histogram percentile of normalized Vp (96) and Suh et al. evaluated parameters derived from the area under the time-signal intensity curve with similar accuracy (97). However, currently there is significant variability in the optimal reported perfusion metric thresholds across institutions and PWI acquisition parameters and analytic methods have not been standardized. DWI has also been evaluated in the posttreatment setting with several studies demonstrating significantly lower ADC values in tumor when compared to treatment changes, most likely attributable to the higher cellularity of tumor (98–100).

However, regardless of the technique employed, an admixture of tumor and treatment change is routinely observed on pathology, which can speciously skew quantitative analyses. Thus, there has been investigation of multiparameteric approaches to improve sensitivity and specificity. For example, a combination approach of DWI with either DSC or DCE demonstrated improved predictive accuracy compared to any single parameter in several studies (91, 92, 101). On the contrary, Prager et al. found a combined DSC and DWI model was not significantly better than rCBV alone (93). While beyond the scope of this review, it should be noted that the advanced imaging toolbox also includes PET and MR spectroscopy, which can further aid in a multiparametric approach.

## Conclusion

Advanced imaging has an ever increasing role to play in the management of patients with gliomas. Preoperatively, imaging adjuncts like DTI and fMRI can facilitate more efficient surgical mapping procedures and even help determine whether intraoperative mapping is necessary in some instances. Future work related to DTI showed focus on making it more accurate and user-independent; this is of particular import in gliomas where tissue edema and destruction can alter white matter pathways in unpredictable ways. Future directions for fMRI may include its use as an adjunct in planning radiation doses to minimize the effects of radiation toxicity or its use in planning safe trajectories and heat maps for patients who undergo laser interstitial thermal therapy.

Despite the strengths of anatomic imaging, there is a need for supplementation with other imaging modalities to better guide treatment. Both PWI and DWI are particularly helpful in biopsy target guidance and in distinguishing recurrent tumor from posttreatment changes. PWI produces more robust parameters than DWI, accounting for greater clinical and research interest. Among PWI techniques, DCE has gained more interest recently given its potential advantages over DSC, particularly in the postoperative setting, and is the preferred technique for tumor surveillance at our institution. Though outside the scope of this article, spectroscopy and positron emission tomography can also be of immense value in resolving diagnostic questions and should be used collaboratively as circumstances require. Either alone or in combination, these advanced imaging tools may eventually provide additional prognostic and molecular information especially given the recent data on molecular heterogeneity between contrast enhancing and non-contrast enhancing disease (102). The potential utility of PWI and DWI will also likely continue to expand as use of novel treatments such as immunotherapy, which can also result in pseudoprogression, become more widely adopted. However, there remains significant variability in the optimal reported quantitative metric thresholds across institutions and neither the RANO criteria for conventional therapies nor the iRANO criteria for immunotherapies have incorporated these techniques to date. Continued research efforts and standardization of acquisition parameters and analytic methods possibly with automation are required to arrive at the most effective approach that can be applied across institutions.

## Author Contributions

GS, LH, PP, RR, RM, and AT have all contributed to this article.

## Conflict of Interest Statement

The authors declare that the research was conducted in the absence of any commercial or financial relationships that could be construed as a potential conflict of interest.
